# Impact of integration of sexual and reproductive health services on consultation duration times: results from the Integra Initiative

**DOI:** 10.1093/heapol/czx141

**Published:** 2017-11-24

**Authors:** Mariana Siapka, Carol Dayo Obure, Susannah H Mayhew, Sedona Sweeney, Justin Fenty, Integra Initiative, Anna Vassall

**Affiliations:** 1 Department of Global Health and Development, Faculty of Public Health and Policy, London School of Hygiene and Tropical Medicine, 15-17 Tavistock Place, London WC1H 9SH, UK; 2 Human Capital Youth and Skills Development Department, African Development Bank, Abidjan, Côte d'Ivoire; 3 London School of Hygiene & Tropical Medicine and; 4 Full list of Integra Initiative team members is provided in the Acknowledgements

**Keywords:** Integration, health systems, family planning, human resources, HIV

## Abstract

The lack of human resources is a key challenge in scaling up of HIV services in Africa’s health care system. Integrating HIV services could potentially increase their effectiveness and optimize the use of limited resources and clinical staff time. We examined the impact of integration of provider initiated HIV counselling and testing (PITC) and family planning (FP counselling and FP provision) services on duration of consultation to assess the impact of PITC and FP integration on staff workload. This study was conducted in 24 health facilities in Kenya under the Integra Initiative, a non-randomized, pre/post intervention trial to evaluate the impact of integrated HIV and sexual and reproductive health services on health and service outcomes. We compared the time spent providing PITC-only services, FP-only services and integrated PITC/FP services. We used log-linear regression to assess the impact of plausible determinants on the duration of clients’ consultation times. Median consultation duration times were highest for PITC-only services (30 min), followed by integrated services (10 min) and FP-only services (8 min). Times for PITC-only and FP-only services were 69.7% higher (95% Confidence Intervals (CIs) 35.8–112.0) and 43.9% lower (95% CIs −55.4 to − 29.6) than times spent on these services when delivered as an integrated service, respectively. The reduction in consultation times with integration suggests a potential reduction in workload. The higher consultation time for PITC-only could be because more pre- and post-counselling is provided at these stand-alone services. In integrated PITC/FP services, the duration of the visit fell below that required by HIV testing guidelines, and service mix between counselling and testing substantially changed. Integration of HIV with FP services may compromise the quality of services delivered and care must be taken to clearly specify and monitor appropriate consultation duration times and procedures during the process of integrating HIV and FP services.

Key Messages

- Integrating HIV services, particularly in resource-limited settings, could potentially increase their effectiveness and optimise the use of limited resources and clinical staff time.
- In Kenya, integration is increasingly being rolled out and significant resources have gone, and are going, into provider initiated HIV counselling and testing (PITC) into FP services at different levels of the health systems. However, the evidence of the impact of integration on staff workloads, and specifically on consultation duration times, remains unclear.
- Integration may improve consultation times for some but not for all services, but most importantly this may mean a reduction in quality for some services.

## Introduction

Global policy recommendations support the integration of HIV and sexual and reproductive health (SRH) (Kennedy *et al.* 2010; Johnson *et al.* 2012). Integration in this context commonly refers to ‘*the delivery of different sets of HIV and SRH services within the same setting, during the same hours, and, preferably, under the same roof, or as part of a facilitated referral within the same facility or to off-site facilities*’ (Liambila *et al.* 2013). The emerging evidence to support the health benefits of integration has identified increased acceptability from both patients and providers; and some potential technical efficiency (‘*providing services or producing outputs at the lowest cost*’) and allocative efficiency (‘*achieving health outcomes at a low cost*’) improvements (Askew and Berer 2003; Sweeney *et al.* 2012, 2014; Obure *et al.* 2015). Specifically, as integration is occurring in the context of a shortage of human resources for health; it is argued that integrating HIV and SRH services can improve the use of limited staff time (World Health Organization and UNAIDS 2006; Callaghan *et al.* 2010; Howard and El-Sadr 2010; Sweeney *et al.* 2014; Siapka *et al.* 2014), and reduce workload (Sweeney *et al.* 2012).

HIV and SRH service integration is therefore increasingly being rolled out in many countries as a mechanism for both increasing demand (through exposure), meeting dual FP and HIV/STI needs and improving efficiency. In Kenya, family planning (FP) and HIV counselling and testing (HCT) services have traditionally been provided separately. The Integra Initiative conduced a household survey among men and women aged 18–49 that was conducted in 2009 (Mak *et al.* 2013). In the two study Provinces (Central and Eastern), the survey showed that, at the population level, 53% women and 67% men had not been tested for HIV or have not received their results in the past year. Furthermore, the survey found there were substantial unmet needs among men and women for both FP and HIV/STI services and that there were significant missed opportunities to provide integrated services to men and women who already attended services but did not receive services that addressed both their FP and HIV/STI prevention and care needs (Mak *et al.* 2013).

While the substantial investment in integration has demonstrated improvements in the uptake of PITC services in Kenya (Kimani *et al.* 2015), there is less known about the impact of these increased demand on human resources. Human resources for health remain scarce in Kenya and elsewhere, but the evidence of the impact of integration on staff workloads remains unclear. Theoretically, integration could reduce the time taken to deliver services, as certain tasks do not need to be repeated. However, integration may also increase providers’ workload, as clients seek and receive more comprehensive services (Baumgartner *et al.* 2014). Where there is excess capacity, an increase in workload may not be problematic, but where staff are already overworked, this could place a strain on scarce resources (Sweeney *et al.* 2014). In this article, we present detailed evidence on the impact of integration of provider initiated HCT (PITC) and FP (FP counselling and FP provision) services on consultation duration times in Kenya, in order to explore how integration may impact overall workload and service quality.

## Methods

We measured and compared consultation duration times for integrated and non-integrated services for a range of different providers. FP consultations were carried out by FP-trained nurses of different cadres; PITC consultations were usually carried out by general nurses, but in some facilities pharmacists or trained HIV counsellors also did this; integrated PITC/FP consultations were carried out by the FP nurses. Currently, there is no guidance on consultation length for integrated services in Kenya and we did not have an a priori hypothesis about the difference in consultation times between integrated PITC/FP services and PITC services and FP services. We therefore compared consultation duration times with one another and to the World Health Organisation (WHO) norms to provide an HIV test at a sufficient quality in terms of providing an accurate results and offering counselling, which is 15 min (World Health Organization 2005). We also analysed the determinants of consultation times using a multivariate analysis, to understand the extent to which integration influenced consultation duration when considering other determinants of consultation time and staff workload.

## Study design and study setting

The study was conducted as part of the Integra Initiative. The study originally sought a controlled, non-randomized intervention design to evaluate the impact of different models of delivering integrated HIV and SRH services on a range of health and service outcomes (Warren *et al.* 2012). As the study took an implementation science approach and was embedded with Ministry of Health activities, however, this design was rendered unreliable over time, as SRH/HIV integration was adopted nationally in Kenya. The Integra Initiative therefore developed a measure of the extent integration to allow for robust assessment of its impact (see Supplementary Annex and Mayhew *et al.* 2016 for rationale, details and measures) on a range of outcomes. Specifically, the Integra Initiative sought to add to the limited evidence base on the benefits of integrating HIV and SRH services, examining service outcomes, quality and costs.

The Integra study sites were purposefully chosen from six priority districts based on previous operational research relationships with the Ministries of Health in Kenya. Integra study sites varied by setting, facility type, ownership and clinical model of integration. In Kenya, the Integra sites included 24 public facilities and 6 non-government organisations (NGO) affiliated SRH clinics. This study uses data collected from the 24 public facilities. The public facilities were selected from two provinces (Central and Eastern) and 6 districts (Nyeri, Nyandarua, Thika, Muranga, Kitui and Makueni) and included a provincial general hospital, 5 district hospitals, 5 sub district hospitals and 13 health centres. The intervention included strengthening integrated delivery within FP and post-natal services as well as broad support for integration to the facility (see Warren *et al.* 2012 and the Supplementary Annex for details). Further details are provided in Supplementary Table S1.

## Study procedure

### Consultation duration times

Data on consultation duration times were measured through client flow assessments (CFAs) (Warren *et al.* 2012). The CFAs were designed using the Patient Flow Analysis, which is a method developed by the Centres for Disease Control to monitor patients’ movements through a clinic over 1 day (Lynam *et al.* 1994; Centers for Disease Control and Prevention 2015). In the Integra evaluation, CFAs were used to measure the duration of consultations and service utilisation patterns among client seeking maternal and child health (MCH) services. CFAs were carried out in all sites over three time points in quarter 3 in 2009, quarter 3 in 2010 and quarter 1 in 2011 covering a period of 5 days (Monday through Friday). A client flow form was provided to all clients accessing MCH services by teams of trained local researchers or service providers. Throughout their visit, each service provider completed the form during the consultation by indicating the session’s start and end times, the service(s) received by the client and any referrals to other providers (Warren *et al.* 2012; Birdthistle *et al.* 2014). Up to five consultations could be recorded. Detailed methods on the client flow component are provided elsewhere, with descriptive analysis of client-receipt of integrated services (Birdthistle *et al.* 2014).

### Integration indicator

Services were grouped into PITC-only, FP-only, integrated PITC/FP and all other services. PITC-only services were the provision of HIV counselling and/or testing services but no FP-related service on the day of the survey; FP-only services were the provision of FP-related services (including counselling, method-provision or method-check); Integrated PITC/FP services were defined as a client receiving both an FP-related service AND an HIV counselling and/or testing service during one of their (up to five) consultations on the day they were surveyed.

The unit of analysis for the client-level dataset was individual clients (who could have multiple consultations in one visit). An integration indicator was constructed for each client, and was described as ‘integrated FP/PITC’, when a combination of FP and PITC services were provided in a single consultation (joined) or when a combination of FP and PITC services were provided in different consultations during the same visit (separate). Together these give an indication of ‘facility level’ (as opposed to provider level) integration. Clients were classified as ‘FP-only’ when at least one of the five consultations was FP and none of the other consultations was PITC. Clients were classified as ‘PITC-only’ when at least one of the five consultations was PITC and none of the other consultations were FP. We estimated a consultation duration time that excluded times for non PITC and FP services. If a person had more than one consultation of the same type the consultation duration times were added to one another to come to a composite consultation duration time.

As a sensitivity analysis we also constructed a provider-level dataset (see Supplementary Material). In this case, the unit of analysis was individual consultations, and a consultation was described as ‘integrated FP/PITC’ when FP and PITC services were provided in the same consultation.

### Determinants of consultation times and staff workload

We examined a range of determinants that were a priori considered plausible to impact on consultation duration times. These included client characteristics of client’s age, sex and type (‘Adult’ indicates an individual going to the consultation alone and ‘adult with child’ indicates an individual going to the consultation with a child); integration characteristics, such as the consultation–integration indicator (described above) and total number of services provided per facility; facility characteristics such as location (urban and rural) and inpatient/outpatient facilities; other geographical/study design characteristics such as province (Central Province and Eastern Province) and the original allocation of the site into intervention or comparison; facility size characteristics, including human resources, such as number of MCH visits per facility at baseline (to estimate size of the facility), total outpatient visits per year per facility, total staff FTE across all services per facility and proportion of clinical staff out of total staff per facility; and workload characteristics, such as facility workload, time of arrival at facility and waiting time.

For some of these metrics we had a choice of methods. Specifically, facility workload was measured as the ratio of the actual staffing levels to the estimated staffing requirements, using an adaptation of basic methods from the WHO’s Workload Indicators of Staffing Needs (WISN). We estimated the time required to deliver services at the facility level through a mixed methods approach to staff time observation; this is an improvement on the typical WISN methodology, which uses expert opinion on timing of health services and may not reflect real practice (Sweeney *et al.* 2014). Time observations were combined with detailed service statistics to estimate the total staff FTE required to deliver services in each facility. Our estimates conservatively assume 220 working days per year accounting for national holidays and leave time, and assume 33% of this time to be taken by administrative duties, trainings and so on—leaving 70 752 annual minutes per clinical staff FTE for direct patient care. For some services, including HCT and HIV care and treatment, we also considered the time of technical staff such as lab technicians and lay counsellors (Shipp 1998; Ozcan and Hornby 1999; World Health Organization 2010; Sweeney *et al.* 2014). A workload ratio greater than 1 (or 100%) indicates some down times for staff members. A ratio of 1 (or 100%) indicates that the estimated time to deliver services is equal to the staff time available for patient visits within a facility. A ratio of < 1 (or 100%) indicates that staff are likely overworked (Sweeney *et al.* 2014). Supplementary Table S1 provides a description of these variables with calculations made, where applicable.

### Sample size

**Figure 1.**
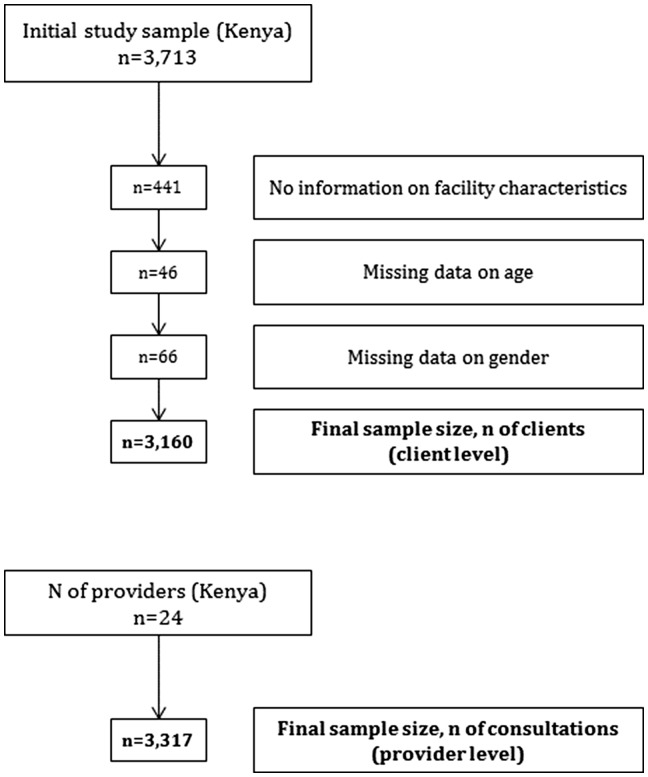
Study sample—flow chart

Of the 13 552 individual clients, 3713 clients had FP-only, PITC-only or PITC/FP consultations and therefore retained for the analysis. Of those, 553 were removed due to lack of information on facility characteristics (*n* = 441), and missing data on age (*n* = 46) and sex (*n* = 66). Final sample sizes were 3160 clients for client-level data and 3317 consultations for provider-level data (Figure 1).

## Data analysis

Descriptive analysis was first conducted for the basic demographic and study characteristics of clients and facilities. When reporting consultation duration times we use medians, interquartiles and range as data were highly skewed. We analysed the differences between consultation duration times for PITC-only, FP-only and integrated services, taking into account other plausible determinants of consultation time, using ordinary least square (OLS) regression analysis with a log transformation for the consultation duration times. We examined differences, or any emerging patterns, in consultation duration times for clients (i.e. client-level data) and individual consultations (provider-level data). We did not have a specific a priori hypothesis about whether and to what extent differences between the two exist. Examining the times for clients, allows us to understand whether this service is more beneficial from a client perspective (in terms of the opportunity cost of time spent receiving a service). Comparing times across consultations is more important from a health provider performance perspective. We run sensitivity analysis on both the client-level and provider-level data to examine the impact of possible measurement bias in consultation duration times by selecting 3 min as the minimum time for any consultation given. All our analyses adjusted for clustering effects at the facility level. For ease of interpretation, regression coefficients were converted into per cent change in consultation duration times using the formula [exp(*β*) − 1]×100. Proportions were transformed into natural logarithmic form, implying that the coefficients of the independent variables can be interested as elasticities (Gerdtham *et al.* 1992). All analyses were carried out in Stata 14.1.

## Results

Facilities substantially varied in terms of overall number of MCH visits (interquartile range 6329–21 686), total outpatient visits (interquartile range 8545–21 804), size of facility (interquartile range 513.8–4189.4 m^2^), staff (interquartile range 21.6–48.6 total FTE) and facility workload (interquartile range 13.1–30.8%) (Table 1).

**Table 1.** Summary statistics for facility characteristics (*n* = 24)

	Mean	SD	Median	Q1; Q3	Range
Integration characteristics					
Total number of services provided per facility	39.7	16.7	41.2	24.9; 51.3	14.2; 79.7
Facility size characteristics					
Total number of mother and child health visits per facility (at baseline in 2009)	14 191	11 974.8	9010	6329; 21 686	2354; 44 840
Total outpatient visits per year per facility	19 064	14 312.0	15 310	8545; 21 804	3805; 52 642
Size of facility (total square meters of consultation rooms)	3409.8	5233.2	894.5	513.8; 4189.4	181.8; 19 542.8
Total staff FTE across all services per facility	33.9	14.9	31.6	21.6; 48.6	6.2; 55.9
Proportion of clinical staff out of total stuff per facility (%)	48.8%	4.3%	49.5%	46.6%; 51.6%	38.6%; 56.8%
Workload characteristics					
Facility workload (at baseline in 2009) (%)	24.9%	17.9%	21.9%	13.1%; 30.8%	3.6%; 84.8%

Table 2 presents summary statistics on clients’ consultation duration times by client and facility characteristics. Mean age of clients was 28 years. Clients’ had a waiting time of 77 min and almost all clients were female (99.6%) (Supplementary Table S2). Median consultation duration times were higher: in the intervention compared to the comparison arm (15 vs 7 min); for PITC-only compared with FP-only consultations (30 vs 8 min) and integrated services PITC/FP (10 min); in the Central Region compared to Eastern Region (15 vs 7 min); and in adults only and adult and child (10 min) compared with unknown coded (8 min) (Table 2). Similar patterns were observed in the provider-level dataset (Supplementary Table S4).

**Table 2.** Summary statistics on clients’ consultation duration times (*n* = 3160)

Variable	Median	Q1; Q3	Range
Integration characteristics			
PITC only	30	15; 53	1; 291
FP only	8	4; 16	1; 268
Integrated PITC & FP ^a^	10	5; 21	1; 183
Integrated PITC & FP (Joined)	9	5; 18	1; 183
Integrated PITC & FP (Separate)	32	23; 44	6; 146
Client characteristics			
*Client type*^b^			
Adult	10	5; 26	1; 291
Adult + child	10	5; 21	1; 268
Unknown	8	5; 15	3; 103
Inpatient/facility characteristics			
*Inpatient/outpatient*			
Inpatient	10	5; 20	1; 291
Outpatient	13	6; 27	1; 159
*Location*			
Rural	10	5; 22	1; 268
Urban	11	5; 26	1; 291
Study selection characteristics			
*Intervention/arm*			
Comparison	7	4; 15	1; 225
Intervention	15	6; 30	1; 291
*FP/PNC model*			
FP model	15	7; 29	1; 256
PNC model	7	4; 15	1; 291
Workload characteristics			
Pooled consultation duration times (min)^c^	10	5; 24	1; 291

FP, Family planning; PITC, Provider-initiated HIV counselling and testing; PNC, Postnatal Care; SD, Standard deviation

a Classified as integrated services when a combination of FP and PITC services were provided in a single consultation (joined) or when a combination of FP and PITC services were used in different consultations (separate). Classified as FP when at least one of the consultations was FP and none of the other consultation were PITC. Classified as PITC when at least one of the consultations was PITC and none of the other consultations were FP

b ‘Adult’ indicates an individual going to the consultation alone, and ‘adult with child’ indicates an individual going to the consultation with a child

c Pooled consultation duration times data of up to five consultations excluding any services not classified as FP, PITC or integrated PITC & FP

Table 3 presents summary statistics by type of service (PITC-only, FP-only and integrated PITC/FP) and facility and patient characteristics. In general, patterns were consistent with median consultation duration times in integrated services being lower when compared with PITC-only but higher when compared with FP-only services irrespective of stratification made by population and facility characteristics. The only exception was when looking at times by client type. Adults with a child had a lower median consultation duration time in integrated services when compared to PITC-only and FP-only (7 vs 20 vs 10 min). Supplementary Table S7 shows the types of services provided in each type of consultation, whether HIV test only, HIV counsel and test and HIV counselling only. PITC only consultations had substantial higher levels of HIV testing (83.2%) and lower levels of counselling (82.4%) than FP/PITC consultations (35.6 and 95.2%, respectively).

**Table 3.** Summary statistics on clients’ consultation duration times^a^ by type of service (*n* = 3160)

	PITC only (*n* = 541)			FP only (*n* = 1897)			**Integrated PITC and FP**^b^**(*n* = 722)**		
	Median	Q1; Q3	Range	Median	Q1; Q3	Range	Median	Q1; Q3	Range
Client characteristics									
*Client type*^c^									
Adult	34	18; 61	2; 291	6	4; 14	1; 219	15	7; 26	1; 168
Adult + child	20	9; 32	1; 159	10	5; 20	1; 268	7	5; 17	1; 183
Unknown	15	.	15; 15	6	4; 9	3; 103	21	10; 32	10; 32
Inpatient/facility characteristics									
*Inpatient/Outpatient*									
Inpatient	27	11; 48	1; 291	8	4; 16	1; 268	7	5; 15	1; 183
Outpatient	31	17; 57	4; 159	8	4; 17	1; 132	18	11; 36	1; 147
*Location*									
Rural	28	15; 55	1; 169	8	4; 16	1; 268	7	5; 16	1; 183
Urban	30	15; 51	2; 291	9	4; 16	1; 256	16	8.5 – 30	2; 137
Study selection characteristics									
*Intervention/Arm*									
Comparison	19	10; 34	1; 225	6	4; 13	1; 162	6	5; 13	1; 183
Intervention	37	22; 60.5	3; 291	10	5; 20	1; 268	20.5	12; 35	1; 147
*FP/PNC model*									
FP model	27	14; 46	2; 180	11	5; 22	1; 256	18	10; 30	1; 146
PNC model	33	15; 68	1; 291	5	4; 10	1; 268	6	4; 12	1; 183

FP, Family planning; PITC, Provider-initiated HIV counselling and testing; PNC, Postnatal Care; SD, Standard deviation.

a Pooled consultation duration times data of up to five consultations excluding any services not classified as FP, PITC or integrated PITC & FP

b Classified as integrated services when a combination of FP and PITC services were provided in a single consultation or, only in the case of individual-level data, when a combination of FP and PITC services were used in different consultations. Classified as FP when at least one of the consultations was FP and none of the other consultation were PITC. Classified as PITC when at least one of the consultations was PITC and none of the other consultations were FP

c ‘Adult’ indicates an individual going to the consultation alone, and ‘adult with child’ indicates an individual going to the consultation with a child

Table 4 presents the results of the OLS regression for log transformed consultation duration times. Our model could explain ∼30% of the variation in consultation duration times, reflecting that there are other potential determinants of consultation times that we had no data on. PITC-only services were significantly related to a 69.7% increase (95% confidence intervals (CIs) 35.8–112.0) in consultation duration times, whereas FP-only services were significantly related to a 43.9% decrease (95% CIs: −55.4 to −29.6) in consultation duration times when compared with integrated services. Total number of services provided per facility (decrease of 0.8% per one added service, 95% CIs −1.5 to −0.1), patient’s age (decrease of 0.6% per one year increase in client’s age, 95% CIs −1.1 to −0.03), intervention arm (increase of 42.8% compared with comparison arm, 95% CIs 11.1–83.5), Eastern Region (decrease of 38.1% compared with Central Region, 95% CIs −55.3 to −14.1), time of arrival (decrease of 0.1% per 1 min increase, 95% CIs −0.12 to −0.02) and proportion of clinical staff (decrease of 1.3% per 1% increase in clinical staff, 95% CIs −2.5 to −0.1) were statistically significantly related to consultation duration times. In sensitivity analysis, the impact of integration on consultation duration times was reduced but remained statistically significant. PITC-only services were significantly related to a 58.5% increase (95% CIs 26.7–98.2) in consultation duration times, whereas FP-only services were significantly related to a 40.5% decrease (95% CIs −51.3 to −27.2) in consultation duration times when compared with integrated services. Direction of effect and size of association remained the same for all plausible determinants, except age (Supplementary Table S9). Similar patterns with less pronounced effects were observed in the provider-level dataset (Supplementary Table S10).

**Table 4.** Impact of plausible determinants on log transformed minutes of clients’ consultation duration times

	Model 1 (*n* = 3160)			Model 2 (*n* = 2929)			Model 3 (*n* = 2491)		
	Beta	% change^a^	95% CIs^a^	Beta	% change^a^	95% CIs^a^	Beta	% change^a^	95% CIs^a^
Integration characteristics									
*Integrated PITC and FP (reference)*									
PITC only	0.558***	74.72	39.70; 118.40	0.485***	62.47	30.09; 102.90	0.018	1.84	−20.98; 31.24
FP only	−0.566***	−43.22	−54.34; −29.36	−0.509***	−39.87	−50.42; −27.08	−1.106***	−66.90	−75.81; −54.70
Total number of services provided per facility	−0.007	−0.70	−1.48; 0.01	−0.009*	−0.91	−1.58; −0.24	−0.003	−0.32	−1.06; 0.42
*Client characteristics*									
Age (years)	−0.006*	−0.60	−1.09; −0.09	−0.004	−0.41	−0.93; 0.10	−0.007*	−0.68	−1.23; −0.12
*Client type*^b^*(Adult—reference)*									
Adult + child	0.042	4.29	−9.99; 20.77	0.029	2.97	−9.52; 17.18	0.066	6.87	−7.28; 23.19
Unknown	−0.241	−21.41	−51.04; 26.01	−0.355	−29.90	−56.70; 13.49	−0.350	−29.56	−59.05; 21.18
Inpatient/facility characteristics									
*Inpatient (reference)*									
Outpatient	0.129	13.77	−17.98; 57.82	0.093	9.70	−17.51; 45.88	0.112	11.81	−19.58; 55.45
Location (Rural – reference)									
Urban	0.535*	70.74	1.93; 186.04	0.369	44.70	−5.13; 120.70	0.385	46.93	−16.17; 157.53
Facility size characteristics									
Total number of mother and child health (MCH) visits per facility (at baseline in 2009) ^c^	0.000	0.00	−0.01; 0.00	0.000	0.00	−0.01; 0.00	0.000	0.00	−0.01; 0.00
Total outpatient visits per year per facility ^d^	0.000	0.00	0.00; 0.00	0.000	0.00	0.00; 0.00	0.000	0.00	0.00; 0.00
Size of facility (total square meters of consultation rooms) ^e^	0.000	0.00	0.00; 0.01	0.000	0.00	0.00; 0.01	0.000	0.00	0.00; 0.01
Total staff FTE across all services per facility	−0.007	−0.70	−1.97; 0.51	−0.007	−0.72	−1.81; 0.39	−0.005	−0.54	−1.97; 0.92
Proportion of clinical staff out of total stuff per facility	−3.920**	−98.02	−99.80; −80.23	−3.548**	−97.12	−99.63; −77.62	−3.828**	−97.83	−99.80; −76.18
Study selection characteristics									
Intervention/Arm (Comparison – reference)									
Intervention	0.396**	48.59	18.56; 86.31	0.353**	42.37	16.07; 74.62	0.315*	36.99	7.15; 75.14
FP/PNC (FP model – reference)									
PNC model	−0.367*	−30.72	−51.65; −0.82	−0.283	−24.63	−43.74; 0.96	−0.331	−28.15	−51.70; 6.88
Workload characteristics									
Facility Workload (at baseline in 2009)	1.189	228.38	−4.21; 1026.47	0.992*	169.53	3.28; 603.43	1.132	210.14	−7.10; 935.44
Time of arrival at facility	−0.001**	−0.10	−0.12; −0.02	−0.001***	−0.08	−0.12; −0.04	−0.001*	−0.07	−0.13; −0.01
Waiting time^f,g^	0.000	0.00	−0.09; 0.09	−0.000	−0.01	−0.09; 0.06	0.000	0.03	−0.07; 0.13
*R*^2^	0.309			0.311			0.307		
AIC	8208			6999			6603		
BIC	8323			7113			6714		

AIC, Akaike Information Criterion; BIC, Bayesian Information Criterion; CIs, Confidence Intervals; FP, Family planning; HR, Human resources; OLS, Ordinary Least Squares; PITC, Provider-initiated HIV counselling and testing; PNC, Postnatal Care

Model 1 using full range of consultation duration times, Model 2 restricted to consultation duration times of over 3 min; Model 3 restricted to separate consultations only

a Regression coefficients were converted into per cent change in consultation duration times using the formula [exp(*β*)−1]×100

b ‘Adult’ indicates an individual going to the consultation alone, and ‘adult with child’ indicates an individual going to the consultation with a child

c Beta  = −0.0000317, % change = −0.003%, 95% CIs = −0.0068% to 0.0005% in Model 1. Beta = −0.0000263, % change = −0.003%, 95% CIs = −0.0055% to 0.0002% in Model 2

d Beta = −0.0000072, % change = −0.001%, 95% CIs = −0.003% to 0.001% in Model 1. Beta = −0.00000631, % change = −0.001%, 95% CIs = −0.002% to 0.001% in Model 2

e Beta = 0.0000373, % change = 0.004%, 95% CIs = −0.001% to 0.009% in Model 1. Beta = 0.0000333, % change = 0.003%, 95% CIs = −0.001% to 0.007% in Model 2

f Beta = 0.0000065, % change = 0.001% in Model 1. Beta = −0.0001351, % change = −0.001%, 95% CIs = −0.004% to 0.001% in Model 2

g Between arrival and first consultation (and subsequent consultations when there was more than one consultation)

* *P* < 0.05, **P < 0.01, ***P < 0.001

## Discussion

The aim of this article was to examine the impact of integration of PITC/FP services on consultation duration times and workload. Using OLS regression with co-variates that were considered to be driving consultation duration times, we found that PITC-only services had higher consultation duration times than for integrated PITC/FP services, and that integrated service consultation duration times were higher than those for FP-only services. The findings around PITC contrast with other studies which have suggested that consultation times of integrated services may be higher when compared with stand-alone services as patients receive a more comprehensive service (Topp *et al.* 2010; Leon *et al.* 2013; Baumgartner *et al.* 2014). In this Journal Supplement, another article examines whether quality of FP care is compromised as a result of integration but finds that it is not (Mutemwa *et al.* 2017)—despite the finding here that integration of FP and PITC does not substantially increase consultation times compared with FP-only consultations. This suggests that the quality of HIV care may be a point of concern. The median consultation times for PITC/FP integrated services in the provider-level analysis were substantially lower (7 min) (at the client level they were slightly higher, 10 min) than times required for accurate HIV-rest results reading (15 min, followed by counselling) (World Health Organization 2005). However, integrated care resulted in higher levels of counselling, but a lower proportion of HIV testing compared with PITC only.

There are several possible explanations for the observed differences in consultation durations for integrated vs non-integrated PITC consultations. The primary explanation is likely to be the different service mix between counselling and testing. While some of this may be due to the differing needs of receiving integrated services—possibly those receiving FP had more recent HIV tests—given the similarities in the demographical profile of those accessing integrated and non-integrated care, it may also be due to provider behaviour in respect of recommending testing. In addition, the higher consultation time for PITC-only compared with integrated PITC/FP services could be due to the fact that more pre-and post-counselling is provided for a PITC-only service compared with the integrated PITC set up within an FP service (Obure *et al.* 2015) (for those clients that receive counselling). Given the association between the proportion of clinical staff at the facility and consultation times, the observed differences in integrated and non-integrated PITC consultation times may also be due to the provision of different services by clinician and non-clinician staff (Hontelez *et al.* 2012), although we were unable to determine that from our data, as we did not have individual level data on the cadre of provider.

Decreases in consultation times may be in part a consequence of a lack of training and support for staff during integration(Johnson *et al.* 2012). Delivering integrated services is likely to require additional and more specialized training than single service provision (Sweeney *et al.* 2014). It should be noted that in this study, clients are receiving both separate and integrated services at the same facility. Johnson *et al.* (2012) reported that health workers may receive training to deliver either HIV or FP services but may not provide either of these services at every consultation. Health workers may also have limited time to provide comprehensive care in all consultations, especially if a daily quota needs to be achieved (Mutemwa *et al.* 2013; Uebel *et al.* 2013). Having said this, it should be noted that integrated care resulted in higher levels of counselling, with fewer tests being provided without counselling. A lack of infrastructure and resources to support integration in some facilities has also been reported, which may impede the full delivery of services(Mutemwa *et al.* 2013; Phiri *et al.* 2016). Other explanations for the lower times for integrated services, include the willingness of health workers to engage in integrated HIV services and provision of care (Uebel *et al.* 2013), in this case carrying out fewer HIV tests. There is also some evidence that the integration of services may increase job satisfaction through improvements in work-based skills and client satisfaction, but at the same time increase workload and stress and reduce the quality of time with clients (Mutemwa *et al.* 2013). These provider-level tensions are further explored in another Integra paper in this Supplement (Mayhew *et al.* 2017).

In applying our findings to policy, these should be considered alongside the research that suggests integration may bring a range of benefits (Johnson *et al.* 2012). In relation to cost-effectiveness, Shade *et al.* (2013) have reported that integration of FP and HIV services can be inexpensive, cost-efficient and cost-effective. Specifically, they found that integration was associated with a marginal cost of $65 for each additional use of more effective FP and $1368 for each pregnancy averted. Reductions in consultation duration times through integration may allow clinics to receive more patients; and without an increase in staff this could lead to reduced client waiting and treatment times overall (Farrell 2007; Mutemwa *et al.* 2013). Obure *et al.* (2015) also reported that better use of human and capital resources can reduce costs of integrated SRH and HIV services.

Some limitations to our analysis should be acknowledged. There may be a risk of information bias in terms of mis-reporting consultation duration times (Sweeney *et al.* 2014). Measurement error cannot be ruled out, as some of the consultation duration times were as low as 1 min and we examined how effect estimates might change using a minimum of 3 min. It might be possible that some of the clients had a pre-test counselling but then referred to do a test elsewhere. In addition, we did not have data on other potentially important determinants of consultation time, such as information on first or a follow-up consultation or contraceptive method used or the type of provider. Client flow data were collected over 1 week, and not on the same week for all sites, which may not be representative of a typical month or annual client flow (Birdthistle *et al.* 2016). Our analysis was undertaken from the provider perspective and we are unable to comment on how duration consultation times may affect quality of care or how patients may value different consultation times (Sweeney *et al.* 2014). Service integration is not unique to HIV and FP services, and future studies should examine how duration times are affected when different services are integrated. In addition, provider time and capacity should be among the many factors that need to be taken into account when selecting an integrated model (Baumgartner *et al.* 2014).

## Conclusion

Integrating HIV services with SRH services like FP has important financial, management and policy implications, particularly in resource-limited settings. Integration may reduce consultation times for some services, but this will depend on the service mix provided. Our findings suggest that the time spent on PITC in an integrated consultation is much lower than in a PITC-only consultation. These findings should also be seen in the context of the additional financial costs and time needed to implement a strategy to integrate services. Future studies should examine the cost-effectiveness of reductions in consultation times for such services, considering the consequences of integration on cost, service mix, quality and service outcomes simultaneously. Going forward, it is important for those implementing integration to have sound systems in place to monitor workload, levels of testing and the quality of care being provided.

## Supplementary Data

Supplementary data are available at *HEAPOL* online.

## Funding

The study was funded by the Bill & Melinda Gates Foundation through the International Planned Parenthood Federation (Grant 48733). The research team is completely independent from the funders. The funders had no role in study design, data collection and analysis, decision to publish or preparation of the manuscript.

*Conflict of interest statement*. None declared.

## Supplementary Material

Supplementary Figure 1

Supplementary Material

Supplementary Tables R2
