# Supplementary figures and images for: Impact of integration of sexual and reproductive health services on consultation duration times: results from the Integra Initiative
Source: Health Policy Plan. 2017 Nov 24;32(Suppl 4):iv82–90. doi: 10.1093/heapol/czx141 (PMC5886289; doi:10.1093/heapol/czx141)

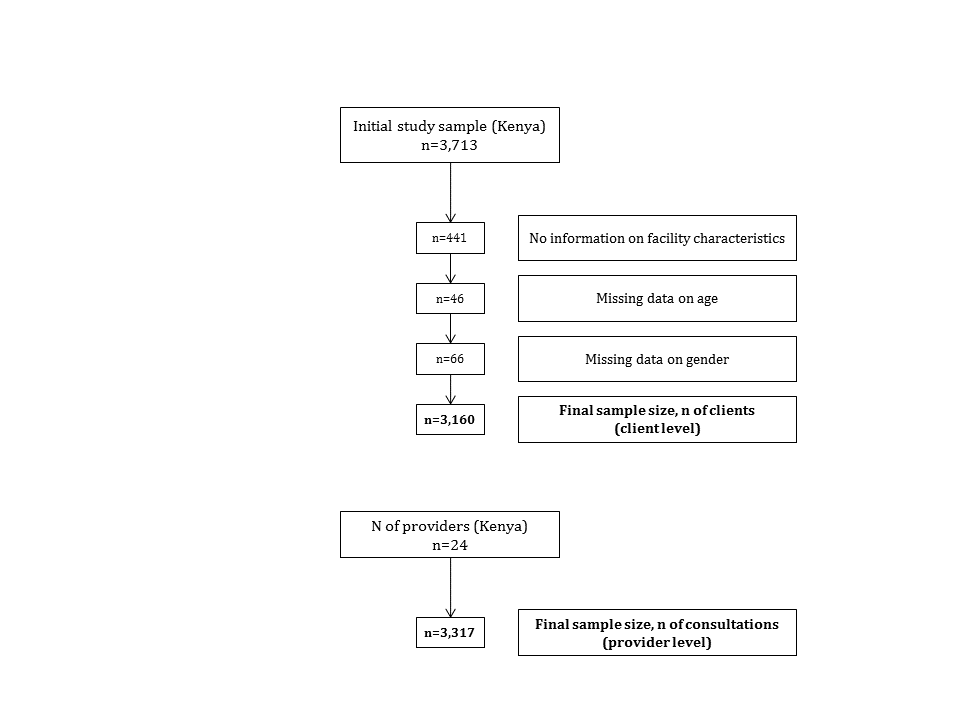

Supplement: Supplementary Figure 1 [file czx141_figure_1_integra_paper.png]
