# Supplementary material for: Impact of integration of sexual and reproductive health services on consultation duration times: results from the Integra Initiative
Source: Health Policy Plan. 2017 Nov 24;32(Suppl 4):iv82–90. doi: 10.1093/heapol/czx141 (PMC5886289; doi:10.1093/heapol/czx141)
Supplement: Supplementary Material [file czx141_supplementary_material_r2.docx]

**Supplementary materials**

***The Integra Initiative***

Integra’s goal was to strengthen the evidence of the benefits and costs of a range of models for delivering HIV services integrated with family planning (FP) and postnatal care (PNC) services in high-prevalence (Swaziland) and medium-prevalence (Kenya) HIV settings. The study originally sought a controlled, non-randomised intervention design to measure the effect of integrated health care (Warren et al 2012). Facilities were assigned, in consultation with the Ministry of Health, to intervention or comparison arms of the study. Intervention facilities received equipment, training on a service-delivery algorithm and a mentorship programme.

The study intervention is described in detail elsewhere ([Warren et al. 2012](#_ENREF_30)), but in short it was implemented between 2009-2011 and was designed, in Kenya, to add the following services into standard FP service delivery: discussion of fertility desires, condom promotion/provision, STI/HIV risk assessment, HIV status check, HTC provision, cervical cancer screening, pre-HIV treatment services and/or referral to HIV treatment unit for HIV+ clients. The provision of these services was supported by training on and the provision of an integrated client counselling toolkit, the ‘Balanced Counseling Strategy Plus’ ([Population Council 2016](#_ENREF_23)). In addition, intervention facilities were supported by nurse/midwife ‘mentors’ who were trained as mentors and provided training on SRH/HIV technical skills and supportive supervision on integrated care (see Ndwiga et al 2014 for details). The layout of some Facilities was also reorganised to support integrated care provision, and essential equipment and supplies were provided to deliver integrated services. By agreement with the Government of Kenya, initial clinical supplies and equipment (including autoclaves) were provided to study facilities to ensure some degree of equity between them at the start of the study. After this, routine government medical supply systems took over (by early 2010). Throughout the study there was regular contact with the MoH.

In Kenya, however, during the trial the government formally adopted and accelerated implementation of integrated HIV and SRH services in all public health facilities (by early 2011). This, together with actions by individual Facility managers, NGOs and external donors, removed operational distinction in service provision between facilities in intervention and comparison arms. Consequently, assessment of the primary outcome was shifted from comparison of study arms to comparison of individual facilities depending on the level of integration each achieved through the study. A facility’s ‘level’ of integration was measured by an “integration index” which gives a relative ranking of Facilities at four timepoints through the study based on aggregate, model-weighted data from a range of indicators (see Mayhew et al 2016 for details).

This paper draws on directly-recorded receipt of integrated services and each facility, therefore the distinction between “intervention” and “comparison” arms is irrelevant. Instead we refer to the Provinces that the study facilities belonged to: Central Province and Eastern Province.

**References**

Warren, C.E., et al., *Study protocol for the Integra Initiative to assess the benefits and costs of integrating sexual and reproductive health and HIV services in Kenya and Swaziland.* BMC Public Health, 2012. **12**: p. 973.

Mayhew, S.H., et al., *Innovation in Evaluating the Impact of Integrated Service-Delivery: The Integra Indexes of HIV and Reproductive Health Integration.* PLoS One, 2016. **11**(1): p. e0146694.

Ndwiga, C., et al., *Exploring experiences in peer mentoring as a strategy for capacity building in sexual reproductive health and HIV service integration in Kenya*. BMC Health Serv Res. 2014. 14:98.

Population Council (2016) *The Balanced Counseling Strategy Plus: A Toolkit for Family Planning Service Providers Working in High HIV/STI Prevalence Settings* (Third Edition). Washington D.C. [cited 2017 27 July]; Available from: <http://www.popcouncil.org/research/the-balanced-counseling-strategy-plus-a-toolkit-for-family-planning-service>

**Table S1. Description of variables included in the models.**

|  | **Variable Description** | **Description of Calculations and formulas** |
| --- | --- | --- |
| **Model** |  |  |
| Family Planning (FP) | In Central Province the focus was on integration of FP and HIV services and entailed performing HIV testing, STI screening and management, cervical cancer screening, condom promotion within FP consultations, as well as active referral to antiretroviral (ART) units for HIV positive FP Clients. | N/A |
| Postnatal Care (PNC) | In Eastern Province the focus was on integration of postnatal care (PNC) and HIV services. This entailed the provision of PNC services to mother and baby, FP services, repeat HIV testing for mother, HIV testing for infant and referral to HIV services for HIV positive mothers and infants as well as referrals for clients requiring additional services. | N/A |
| **Intervention/Comparison arm** | A pair-wise matching sampling design was used to select facilities in the comparison arm based on four criteria: high client load, a minimum of two providers qualified in and currently delivering FP services; a range of services available and no current provision of fully integrated PNC-HIV and FP-HIV services. Comparison facilities were in different districts in the same province. Data were available from twelve public facilities in each arm | N/A |
| **Integration Characteristics** |  |  |
| PITC only | At least one of the five consultations was PITC and none of the other consultations were FP. | N/A |
| FP only | At least one of the five consultations was FP and none of the other consultations were PITC. | N/A |
| Total number of services provided per facility | The number of all the different services provided in each facility | $\mathrm{Total}n of services$ = $\sum_{f} n_{s}$  Where *f* denotes the facility and *s* denotes a specific service provided. |
| **Facility Size Characteristics** |  |  |
| Total number of mother and child health (MCH) visits per facility | The number of mother and child health visits per facility | N/A |
| Total outpatient visits per year per facility | The total number of outpatient visits per year per facility | N/A |
| Size of facility (total square meters of consultation rooms) | The size of facility using the size in square meters of all consultation rooms in the facility. | $\mathrm{Total}n of services$ = $\sum_{f} {size}_{cr}$  Where *f* denotes the facility and *cr* denotes a consultation room. |
| Total staff FTE across all services per facility | Provides the total staff FTE, comprising of clinical, technical and admin staff, for all services for each facility. | $Total staff FTE$ = $\sum_{f} {FTE}_{p}$  Where *f* denotes the facility and *p* denotes the FTE for clinical, technical and admin staff. |
| Proportion of clinical staff out of total staff per facility | Provides the proportion of the number of clinical staff out of the total number of staff in each facility | N/A |
| **Workload Characteristics** |  |  |
| Facility Workload | A ratio of the actual staffing levels to the estimated staffing requirements | By dividing the mean time required over the FTE time required (across all different types of services).  ${Facility workload}_{f}$ = $\frac{t}{FTE}$  Where *f* denotes the facility, *t* denotes the mean time required (actual staffing levels) and *FTE* denotes the FTE time required (estimated staffing requirements). |
| Time of arrival at facility | Time in minutes spend from 12 midnight | N/A |
| Waiting time | The total time duration in minutes of clients between arrival and first consultation (and subsequent consultations when there was more than one consultation). | $Waiting time$ = $\sum_{i} w_{c}$  Where *i* denotes the individual client, *w* denotes the waiting time and *c* denotes the consultation. |
| **Consultation Times** |  |  |
| Client-level data | Unit of analysis was individual clients. Pooled consultation duration times of up to five consultations excluding any services not classified as FP, PITC or integrated PITC & FP. | The composite consultation duration times (CCDT) was generated using the formula below  CCDT = $\sum_{i} t_{c}$  Where *i* denotes the individual client, *t* denotes the consultation time and *c* denotes a consultation that was defined as either FP or PITC in different consultations for the same patient or a combination of the two services in a single consultation for the same patient. |
| Provider-level data | Unit of analysis was individual consultations. A consultation was described as ‘integrated FP/PITC’ when FP and PITC services were provided in the same consultation. | The consultation duration times (CDT) was generated using the formula below  CDT = $\sum t_{c}$  Where *t* denotes the consultation time and *c* denotes a consultation that was defined as FP and PITC services in a single consultation. |

FP: Family planning; FTE: Full-time equivalent; MCH: Mother and child health; N/A: not available; PITC: Provider-initiated HIV counselling and testing; PMTCT: Prevention of Mother to Child Transmission.

**Table S2. Summary statistics in client and provider level data.**

|  | **Client level data (n=3,160)** | | **Provider level data (n=3,317)** | |
| --- | --- | --- | --- | --- |
|  | **N / Mean** | **% / SD** | **N / Mean** | **% / SD** |
| **Integration Characteristics** |  |  |  |  |
| PITC only | 541 | 17.12% | 606 | 18.27% |
| FP only | 1,897 | 60.03% | 2,040 | 61.50% |
| Integrated PITC/FP ^a^ | 722 | 22.85% | 671 | 20.23% |
| Integrated PITC/FP (Joined) | 669 | 21.17% | - | - |
| Integrated PITC/FP (Separate) | 53 | 1.68% | - | - |
| **Client Characteristics** |  |  |  |  |
| Age (years) | 28 | 7.0 | 28 | 6.9 |
| Client type  ^b^ |  |  |  |  |
| Adult | 1,736 | 54.94% | 1,827 | 55.08% |
| Adult + child | 1,413 | 44.72% | 1,478 | 44.56% |
| Unknown | 11 | 0.25% | 12 | 0.36% |
| Gender |  |  |  |  |
| Male | 11 | 0.35% | 12 | 0.36%) |
| Female | 3,149 | 99.65% | 3,305 | 99.64% |
| **Inpatient/Facility Characteristics** |  |  |  |  |
| Inpatient/Outpatient |  |  |  |  |
| Inpatient | 2,069 | 65.47% | 2,145 | 64.67% |
| Outpatient | 1,091 | 34.53% | 1,172 | 35.33% |
| Location |  |  |  |  |
| Rural | 2,085 | 65.98% | 2,192 | 66.08% |
| Urban | 1,075 | 34.02% | 1,125 | 33.92% |
| **Study Selection Characteristics** |  |  |  |  |
| Intervention/Arm |  |  |  |  |
| Comparison | 1,556 | 49.24% | 1,617 | 48.75% |
| Intervention | 1,604 | 50.76% | 1,700 | 51.25% |
| Region |  |  |  |  |
| Central Province | 1,731 | 54.78% | 1,829 | 55.14% |
| Eastern Province | 1,429 | 45.22% | 1,488 | 44.86% |
| **Workload Characteristics** |  |  |  |  |
| Waiting times (mins) ^c^ | 77 | 73.3 | 77 | 73.1 |
| Time of arrival at facility ^d^ | 653 | 100.4 | 653 | 99.8 |

FP: Family planning; PITC: Provider-initiated HIV counselling and testing; PNC: Postnatal Care; SD: Standard deviation.

^a^: Classified as integrated services when a combination of FP and PITC services were provided in a single consultation (joined) or when a combination of FP and PITC services were used in different consultations (separate). Classified as FP when at least one of the consultations was FP and none of the other consultation were PITC. Classified as PITC when at least one of the consultations was PITC and none of the other consultations were FP.

^b^: ‘Adult’ indicates an individual going to the consultation alone, and ‘adult with child’ indicates an individual going to the consultation with a child.

^c^: Between arrival and first consultation (and subsequent consultations when there was more than one consultation).

^d^: Time in minutes spend from 12 midnight.

**Table S3. Summary statistics in client and provider level data by type of service.**

|  | **Client-level dataset (n=3,160)** | | | | | | **Provider-level dataset (n=3,317)** | | | | | |
| --- | --- | --- | --- | --- | --- | --- | --- | --- | --- | --- | --- | --- |
|  | **PITC only**  **(n=541)** | | **FP only**  **(n=1,897)** | | **Integrated PITC/FP ^a^**  **(n=722)** | | **PITC only**  **(n=606)** | | **FP only**  **(n=2,040)** | | **Integrated PITC/FP ^a^**  **(n=671)** | |
|  | N / Mean | % / SD | N / Mean | % / SD | N / Mean | % / SD | N / Mean | % / SD | N / Mean | % / SD | N / Mean | % / SD |
| **Client Characteristics** |  |  |  |  |  |  |  |  |  |  |  |  |
| Age (years) | 27 | 7.5 | 28 | 6.9 | 28 | 6.6 | 27 | 7.5 | 28 | 6.9 | 28 | 6.5 |
| Client type ^b^ |  |  |  |  |  |  |  |  |  |  |  |  |
| Adult | 379 | 21.83% | 1,040 | 59.91% | 317 | 18.26% | 424 | 23.21% | 1122 | 61.41% | 281 | 15.38% |
| Adult + child | 161 | 11.39% | 849 | 60.08% | 403 | 28.52% | 181 | 12.25% | 909 | 61.50% | 388 | 26.25% |
| Unknown | 1 | 9.09% | 8 | 72.73% | 2 | 18.18% | 1 | 8.33% | 9 | 75.00% | 2 | 16.67% |
| Gender |  |  |  |  |  |  |  |  |  |  |  |  |
| Male | 10 | 90.91% | 1 | 9.09% | 0 | 0.00% | 11 | 91.67% | 1 | 8.33% | 0 | 0.00% |
| Female | 531 | 16.86% | 1,896 | 60.21% | 722 | 22.93% | 595 | 18.00% | 2039 | 61.69% | 671 | 20.30% |
| **Inpatient/Facility Characteristics** |  |  |  |  |  |  |  |  |  |  |  |  |
| Inpatient/Outpatient |  |  |  |  |  |  |  |  |  |  |  |  |
| Inpatient | 314 | 15.18% | 1,218 | 58.87% | 537 | 25.95% | 332 | 15.48% | 1,286 | 59.95% | 527 | 24.57% |
| Outpatient | 227 | 20.81% | 679 | 62.24% | 185 | 16.96% | 274 | 23.38% | 754 | 64.33% | 144 | 12.29% |
| Location |  |  |  |  |  |  |  |  |  |  |  |  |
| Rural | 351 | 16.83% | 208 | 57.94% | 526 | 25.23% | 408 | 18.61% | 1306 | 59.58% | 478 | 21.81% |
| Urban | 190 | 17.67% | 689 | 64.09% | 196 | 18.23% | 198 | 17.60% | 734 | 65.24% | 193 | 17.16% |
| **Study Selection Characteristics** |  |  |  |  |  |  |  |  |  |  |  |  |
| Intervention/Arm |  |  |  |  |  |  |  |  |  |  |  |  |
| Comparison | 229 | 14.72% | 833 | 53.53% | 494 | 31.75% | 243 | 15.03% | 886 | 54.79% | 488 | 30.18% |
| Intervention | 312 | 19.45% | 1,064 | 66.33% | 228 | 14.21% | 363 | 21.35% | 1,154 | 67.88% | 183 | 10.76% |
| Region |  |  |  |  |  |  |  |  |  |  |  |  |
| Central Province | 345 | 19.93% | 1,098 | 63.43% | 288 | 16.64% | 378 | 20.67% | 1,190 | 65.06% | 261 | 14.27% |
| Eastern Province | 196 | 13.72% | 799 | 55.91% | 434 | 30.37% | 228 | 15.32% | 850 | 57.12% | 410 | 27.55% |
| **Workload Characteristics** |  |  |  |  |  |  |  |  |  |  |  |  |
| Waiting times (mins) ^c^ | 96 | 77.1 | 74 | 75.3 | 71 | 61.6 | 94 | 76.5 | 74 | 74.9 | 70 | 61.5 |
| Time of arrival at facility ^d^ | 626 | 93.3 | 649 | 101.3 | 683 | 95.4 | 630 | 94.1 | 650 | 100.3 | 685 | 95.8 |

FP: Family planning; PITC: Provider-initiated HIV counselling and testing; PNC: Postnatal Care; SD: Standard deviation

^a^: Classified as integrated services when a combination of FP and PITC services were provided in a single consultation or, only in the case of individual-level data, when a combination of FP and PITC services were used in different consultations. Classified as FP when at least one of the consultations was FP and none of the other consultation were PITC. Classified as PITC when at least one of the consultations was PITC and none of the other consultations were FP.

^b^: ‘Adult’ indicates an individual going to the consultation alone, and ‘adult with child’ indicates an individual going to the consultation with a child.

^c^: Between arrival and first consultation (and subsequent consultations when there was more than one consultation).

^d^: Time in minutes spend from 12 midnight.

Table S4. Summary statistics on consultation duration times in the provider-level dataset (n=3,317).

|  | **Median** | **Q1-Q3** | **Range** |
| --- | --- | --- | --- |
| **Integration Characteristics** |  |  |  |
| PITC only | 21 | 10; 34 | 1; 141 |
| FP only | 6 | 4; 13 | 1; 268 |
| Integrated PITC/FP  ^a^ | 7 | 5; 15 | 1; 183 |
| **Client Characteristics** |  |  |  |
| Client type |  |  |  |
| Adult | 10 | 5; 20 | 1; 219 |
| Adult + child  ^b^ | 7 | 4; 15 | 1; 268 |
| Unknown | 6 | 4; 10 | 2; 103 |
| **Inpatient/Facility Characteristics** |  |  |  |
| Inpatient/Outpatient |  |  |  |
| Inpatient | 7 | 4; 15 | 1; 268 |
| Outpatient | 10 | 5; 21 | 1; 147 |
| Location |  |  |  |
| Rural | 8 | 5; 17 | 1; 268 |
| Urban | 9 | 5; 17 | 1; 155 |
| **Study Selection Characteristics** |  |  |  |
| Intervention/Arm |  |  |  |
| Comparison | 6 | 4; 13 | 1; 183 |
| Intervention | 10 | 5; 23 | 1; 268 |
| Region |  |  |  |
| Central Province | 10 | 5; 21 | 1; 219 |
| Eastern Province | 6 | 4; 13 | 1; 268 |
| **Workload Characteristics** |  |  |  |
| Consultation duration times (mins) | 8 | 5; 17 | 1; 268 |

FP: Family planning; PITC: Provider-initiated HIV counselling and testing; PNC: Postnatal Care; SD: Standard deviation.

^a^: Classified as integrated services when a combination of FP and PITC services were provided in a single consultation or when a combination of FP and PITC services were used in different consultations. Classified as FP when at least one of the consultations was FP and none of the other consultation were PITC. Classified as PITC when at least one of the consultations was PITC and none of the other consultations were FP.

^b^: ‘Adult’ indicates an individual going to the consultation alone, and ‘adult with child’ indicates an individual going to the consultation with a child.

**Table S5. Number of consultations per facility by type of service.**

| **Facility ID** | **Integrated PITC/FP** | **PICT only** | **FP only** | **Total** |
| --- | --- | --- | --- | --- |
| site001 | 0 | 34 | 171 | 205 |
| site002 | 76 | 49 | 137 | 262 |
| site003 | 21 | 58 | 70 | 149 |
| site004 | 1 | 23 | 255 | 279 |
| site005 | 28 | 54 | 98 | 180 |
| site006 | 28 | 20 | 44 | 92 |
| site007 | 26 | 45 | 31 | 102 |
| site008 | 48 | 15 | 78 | 141 |
| site009 | 7 | 23 | 67 | 97 |
| site010 | 1 | 5 | 53 | 59 |
| site011 | 4 | 45 | 111 | 160 |
| site012 | 21 | 7 | 75 | 103 |
| site013 | 6 | 15 | 151 | 172 |
| site014 | 2 | 29 | 34 | 65 |
| site015 | 9 | 5 | 43 | 57 |
| site016 | 0 | 15 | 59 | 74 |
| site017 | 11 | 22 | 31 | 64 |
| site018 | 1 | 39 | 61 | 101 |
| site019 | 37 | 40 | 166 | 243 |
| site020 | 322 | 28 | 60 | 410 |
| site021 | 2 | 12 | 27 | 41 |
| site022 | 8 | 11 | 88 | 107 |
| site023 | 0 | 1 | 65 | 66 |
| site024 | 12 | 11 | 65 | 88 |
| Total | 671 | 606 | 2,040 | 3,317 |

**Table S6. Referrals across consultations by type of service.**

|  | **Integrated PITC/FP** | | **PICT only** | | **FP only** | | **Total** | |
| --- | --- | --- | --- | --- | --- | --- | --- | --- |
|  | **n** | **%** | **n** | **%** | **n** | **%** | **n** | **%** |
| **Consultation 1** |  |  |  |  |  |  |  |  |
| FP counselling |  |  |  |  |  |  |  |  |
| No | 708 | 98.06% | 538 | 99.45% | 1,832 | 96.62% | 3,078 | 97.44% |
| Yes | 14 | 1.94% | 3 | 0.55% | 64 | 3.38% | 81 | 2.56% |
| FP provision |  |  |  |  |  |  |  |  |
| No | 691 | 95.71% | 541 | 100.00% | 1,807 | 95.26% | 3,039 | 96.17% |
| Yes | 31 | 4.29% | 0 | 0.00% | 90 | 4.74% | 121 | 3.83% |
| HIV counselling |  |  |  |  |  |  |  |  |
| No | 700 | 96.95% | 489 | 90.39% | 1,887 | 99.47% | 3,076 | 97.34% |
| Yes | 22 | 3.05% | 52 | 9.61% | 10 | 0.53% | 84 | 2.66% |
| HIV testing |  |  |  |  |  |  |  |  |
| No | 695 | 96.26% | 469 | 86.69% | 1,888 | 99.53% | 3,052 | 96.58% |
| Yes | 27 | 3.74% | 72 | 13.31% | 9 | 0.47% | 108 | 3.42% |
| **Consultation 2** |  |  |  |  |  |  |  |  |
| FP counselling |  |  |  |  |  |  |  |  |
| No | 297 | 97.06% | 393 | 99.24% | 639 | 99.38% | 1,329 | 98.81% |
| Yes | 9 | 2.94% | 3 | 0.76% | 4 | 0.62% | 16 | 1.19% |
| FP provision |  |  |  |  |  |  |  |  |
| No | 283 | 92.48% | 395 | 99.75% | 635 | 98.76% | 1,313 | 97.62% |
| Yes | 23 | 7.52% | 1 | 0.25% | 8 | 1.24% | 32 | 2.38% |
| HIV counselling |  |  |  |  |  |  |  |  |
| No | 305 | 99.67% | 388 | 97.98% | 642 | 99.84% | 1,335 | 99.26% |
| Yes | 1 | 0.33% | 8 | 2.02% | 1 | 0.16% | 10 | 0.74% |
| HIV testing |  |  |  |  |  |  |  |  |
| No | 305 | 99.67% | 387 | 97.73% | 642 | 99.84% | 1,334 | 99.18% |
| Yes | 1 | 0.33% | 9 | 2.27% | 1 | 0.16% | 11 | 0.82% |
| **Consultation 3** |  |  |  |  |  |  |  |  |
| FP counselling |  |  |  |  |  |  |  |  |
| No | 209 | 99.52% | 259 | 100.00% | 427 | 99.30% | 895 | 99.56% |
| Yes | 1 | 0.48% | 0 | 0.00% | 3 | 0.70% | 4 | 0.44% |
| FP provision |  |  |  |  |  |  |  |  |
| No | 207 | 98.57% | 259 | 100.00% | 426 | 99.07% | 892 | 99.22% |
| Yes | 3 | 1.43% | 0 | 0.00% | 4 | 0.93% | 7 | 0.78% |
| HIV counselling |  |  |  |  |  |  |  |  |
| No | 209 | 99.52% | 256 | 98.84% | 430 | 100.00% | 895 | 99.56% |
| Yes | 1 | 0.48% | 3 | 1.16% | 0 | 0.00% | 4 | 0.44% |
| HIV testing |  |  |  |  |  |  |  |  |
| No | 210 | 100.00% | 256 | 98.84% | 430 | 100.00% | 896 | 99.67% |
| Yes | 0 | 0.00% | 3 | 1.16% | 0 | 0.00% | 3 | 0.33% |
| **Consultation 4** |  |  |  |  |  |  |  |  |
| FP counselling |  |  |  |  |  |  |  |  |
| No | 177 | 100.00% | 197 | 100.00% | 401 | 100.00% | 775 | 100.00% |
| FP provision |  |  |  |  |  |  |  |  |
| No | 177 | 100.00% | 197 | 100.00% | 401 | 100.00% | 775 | 100.00% |
| HIV counselling |  |  |  |  |  |  |  |  |
| No | 177 | 100.00% | 197 | 100.00% | 401 | 100.00% | 775 | 100.00% |
| HIV testing |  |  |  |  |  |  |  |  |
| No | 177 | 100.00% | 197 | 100.00% | 401 | 100.00% | 775 | 100.00% |
| **Consultation 5** |  |  |  |  |  |  |  |  |
| FP counselling |  |  |  |  |  |  |  |  |
| No | 174 | 100.00% | 191 | 100.00% | 396 | 100.00% | 761 | 100.00% |
| FP provision |  |  |  |  |  |  |  |  |
| No | 174 | 100.00% | 191 | 100.00% | 396 | 100.00% | 761 | 100.00% |
| HIV counselling |  |  |  |  |  |  |  |  |
| No | 174 | 100.00% | 191 | 100.00% | 396 | 100.00% | 761 | 100.00% |
| HIV testing |  |  |  |  |  |  |  |  |
| No | 174 | 100.00% | 191 | 100.00% | 396 | 100.00% | 761 | 100.00% |

**Table S7. Distribution of HIV counselling and testing services by type of service.**

|  | **Integrated PITC/FP** | | **PITC only** | |
| --- | --- | --- | --- | --- |
|  | **n** | **%** | **n** | **%** |
| **Consultation 1** |  |  |  |  |
| No HIV counselling or HIV testing | 0 | 0.0% | 0 | 0.0% |
| HIV counselling only | 404 | 67.3% | 82 | 20.3% |
| HIV testing only | 29 | 4.8% | 59 | 14.6% |
| HIV counselling and testing | 167 | 27.8% | 262 | 65.0% |
| **Consultation 2** |  |  |  |  |
| No HIV counselling or HIV testing | 0 | 0.0% | 0 | 0.0% |
| HIV counselling only | 25 | 40.3% | 15 | 8.6% |
| HIV testing only | 3 | 4.8% | 44 | 25.1% |
| HIV counselling and testing | 34 | 54.8% | 116 | 66.3% |
| **Consultation 3** |  |  |  |  |
| No HIV counselling or HIV testing | 0 | 0.0% | 0 | 0.0% |
| HIV counselling only | 3 | 33.3% | 5 | 20.8% |
| HIV testing only | 0 | 0.0% | 3 | 12.5% |
| HIV counselling and testing | 6 | 66.7% | 16 | 66.7% |
| **Consultation 4** |  |  |  |  |
| No HIV counselling or HIV testing | 0 | 0.0% | 0 | 0.0% |
| HIV counselling only | 1 | 100.0% | 1 | 12.5% |
| HIV testing only | 0 | 0.0% | 2 | 25.0% |
| HIV counselling and testing | 0 | 0.0% | 5 | 62.5% |
| **Consultation 5** |  |  |  |  |
| No HIV counselling or HIV testing | 0 | 0.0% | 0 | 0.0% |
| HIV counselling and testing | 0 | 0.0% | 3 | 100.0% |
| **All consultations** |  |  |  |  |
| No HIV counselling or HIV testing | 0 | 0.0% | 0 | 0.0% |
| HIV counselling only | 433 | 64.4% | 103 | 16.8% |
| HIV testing only | 32 | 4.8% | 108 | 17.6% |
| HIV counselling and testing | 207 | 30.8% | 402 | 65.6% |

Table S8. Summary statistics of consultation duration times by type of service (n=3,317)

|  | **PITC only (n=606)** | | | **FP only (n=2,040)** | | | **Integrated PITC/FP ^a^ (n=671)** | | |
| --- | --- | --- | --- | --- | --- | --- | --- | --- | --- |
|  | Median | Q1; Q3 | Range | Median | Q1; Q3 | Range | Median | Q1; Q3 | Range |
| **Client Characteristics** |  | | |  | | |  | | |
| Client type ^b^ |  |  |  |  |  |  |  |  |  |
| Adult | 25 | 14; 39 | 1; 141 | 6 | 4; 12 | 1; 219 | 11 | 6; 20 | 1; 147 |
| Adult + child | 15 | 5; 25 | 1; 140 | 7 | 5; 14 | 1; 268 | 5 | 4; 11 | 1; 183 |
| Unknown | 15 | . | 15; 15 | 5 | 3; 8 | 2; 103 | 7.5 | 5; 10 | 5; 10 |
| **Inpatient/Facility Characteristics** |  |  |  |  |  |  |  |  |  |
| Inpatient/Outpatient |  |  |  |  |  |  |  |  |  |
| Inpatient | 20 | 10; 31 | 1; 141 | 6 | 4; 12 | 1; 268 | 5 | 4; 14 | 1; 183 |
| Outpatient | 24 | 14; 39 | 1; 140 | 7 | 4; 14 | 1; 125 | 14.5 | 9; 23.5 | 1; 147 |
| Location |  |  |  |  |  |  |  |  |  |
| Rural | 21 | 10; 34 | 1; 140 | 7 | 4; 14 | 1; 268 | 5 | 4; 13 | 1; 183 |
| Urban | 21 | 10; 34 | 1; 141 | 6 | 4; 11 | 1; 155 | 13 | 7; 25 | 2; 70 |
| **Study Selection Characteristics** |  |  |  |  |  |  |  |  |  |
| Intervention/Arm |  |  |  |  |  |  |  |  |  |
| Comparison | 15 | 6; 25 | 1; 141 | 5 | 4; 10 | 1; 155 | 5 | 4; 11 | 1; 183 |
| Intervention | 25 | 15; 40 | 2; 135 | 7 | 5; 14 | 1; 268 | 15 | 10; 26 | 1; 147 |
| Region |  |  |  |  |  |  |  |  |  |
| Central Province | 21 | 10; 32 | 1; 141 | 8 | 5; 15 | 1; 219 | 15 | 8; 25 | 1; 89 |
| Eastern Province | 21.5 | 14; 37 | 1; 135 | 5 | 4; 9 | 1; 268 | 5 | 4; 10 | 1; 183 |

FP: Family planning; PITC: Provider-initiated HIV counselling and testing; PNC: Postnatal Care; SD: Standard deviation

^a^: Classified as integrated services when a combination of FP and PITC services were provided in a single consultation or, only in the case of individual-level data, when a combination of FP and PITC services were used in different consultations. Classified as FP when at least one of the consultations was FP and none of the other consultation were PITC. Classified as PITC when at least one of the consultations was PITC and none of the other consultations were FP.

^b^: ‘Adult’ indicates an individual going to the consultation alone, and ‘adult with child’ indicates an individual going to the consultation with a child.

**Table S9. Impact of plausible determinants on log transformed minutes of clients’ consultation duration times (n=2,929).**

| **Integration Characteristics** | **beta** | **% change**^a^ | **95% CIs**^a^ |
| --- | --- | --- | --- |
| Integrated PITC/FP (reference) |  |  |  |
| PITC only | 0.460^***^ | 58.46 | 26.69; 98.19 |
| FP only | -0.519^***^ | -40.48 | -51.33; -27.22 |
| Total number of services provided per facility | -0.010^**^ | -1.00 | -1.65; -0.35 |
| **Client Characteristics** |  |  |  |
| Age (years) | -0.004 | -0.38 | -0.91; 0.15 |
| Client type ^b^ (Adult – reference) |  |  |  |
| Adult + child | 0.026 | 2.64 | -9.68; 16.64 |
| Unknown | -0.346 | -29.25 | -56.29; 14.54 |
| **Inpatient/Facility Characteristics** |  |  |  |
| Inpatient (Reference) |  |  |  |
| Outpatient | -0.010 | -0.96 | -22.99; 27.36 |
| Location (Rural – reference) |  |  |  |
| Urban | 0.089 | 9.27 | -25.63; 60.54 |
| **Facility Size Characteristics** |  |  |  |
| Total number of mother and child health (MCH) visits per facility (at baseline in 2009) ^c^ | 0.000 | 0.00 | 0.00; 0.00 |
| Total outpatient visits per year per facility ^d^ | 0.000 | 0.00 | 0.00; 0.00 |
| Size of facility (total square meters of consultation rooms) ^e^ | 0.000 | 0.00 | 0.00; 0.01 |
| Total staff FTE across all services per facility | -0.014 | -1.42 | -3.17; 0.37 |
| Proportion of clinical staff out of total stuff per facility | -1.207^*^ | -1.21 | -2.26; -0.16 |
| **Study Selection Characteristics** |  |  |  |
| Intervention/Arm (Comparison – reference) |  |  |  |
| Intervention | 0.323^**^ | 38.14 | 10.46; 72.77 |
| Region (Central Province – reference) |  |  |  |
| Eastern Province | -0.372^**^ | -31.07 | -47.45; -9.59 |
| **Workload Characteristics** |  |  |  |
| Facility Workload (at baseline in 2009) | 0.043 | 0.04 | -0.31; 0.40 |
| Time of arrival at facility | -0.001^***^ | -0.08 | -0.12; -0.04 |
| Waiting time ^f, g^ | 0.000 | -0.01 | -0.09; 0.06 |
| **R^2^** | 0.305 |  |  |
| **AIC** | 7,024 |  |  |
| **BIC** | 7,137 |  |  |

AIC: Akaike Information Criterion; BIC: Bayesian Information Criterion; CIs: Confidence Intervals; FP: Family planning; HR: Human resources; OLS: Ordinary Least Squares; PITC: Provider-initiated HIV counselling and testing; PNC: Postnatal Care.

Model restricted to consultation duration times of over 3 minutes.

^a^: Regression coefficients were converted into per cent change in consultation duration times using the formula [exp(β)−1]100. Proportion of clinical staff out of total stuff per facility and facility workload were log transformed and treated as elasticities.

^b^: ‘Adult’ indicates an individual going to the consultation alone, and ‘adult with child’ indicates an individual going to the consultation with a child.

^c^: Beta = -0.000017077, % change = -0.002%, 95% CIs = -0.0049% to 0.0014%.

^d^: Beta = 0.000004914, % change = 0.001%, 95% CIs = -0.0018% to 0.0028%.

^e^: Beta = 0.000016558, % change = 0.002%, 95% CIs = -0.0034% to 0.0067%.

^f^: Beta = -0.000144338, % change = -0.001%.

^g^: Between arrival and first consultation (and subsequent consultations when there was more than one consultation).

^*^ *p* < 0.05, ^**^ *p* < 0.01, ^***^ *p* < 0.001.

**Table S10. Impact of plausible determinants on log transformed minutes of consultation duration times.**

|  | **Model 1 (n=3,317)** | | | **Model 2 (n=2,996)** | | |
| --- | --- | --- | --- | --- | --- | --- |
|  | **beta** | **% change**^a^ | **95% CIs**^a^ | **beta** | **% change**^a^ | **95% CIs**^a^ |
| **Integration Characteristics** |  |  |  |  |  |  |
| Integrated PITC/FP (reference) |  |  |  |  |  |  |
| PITC only | 0.363^***^ | 43.74 | 15.92; 78.23 | 0.309^**^ | 36.23 | 12.81; 64.50 |
| FP only | -0.554^***^ | -42.54 | -54.80; -26.97 | -0.476^***^ | -37.88 | -48.23; -25.47 |
| Total number of services provided per facility | -0.006 | -0.59 | -1.19; 0.01 | -0.007^**^ | -0.72 | -1.26; -0.19 |
| **Client Characteristics** |  |  |  |  |  |  |
| Age (years) | -0.003 | -0.32 | -0.74; 0.11 | -0.003 | -0.27 | -0.69; 0.15 |
| Client type ^b^ (Adult – reference) |  |  |  |  |  |  |
| Adult + child | -0.103 | -9.75 | -22.19; 4.68 | -0.096 | -9.14 | -20.76; 4.17 |
| Unknown | -0.259 | -22.85 | -54.10; 29.67 | -0.335 | -28.47 | -57.61; 20.72 |
| **Inpatient/Facility Characteristics** |  |  |  |  |  |  |
| Inpatient (Reference) |  |  |  |  |  |  |
| Outpatient | 0.119 | 12.66 | -15.57; 50.34 | 0.070 | 7.30 | -13.70; 33.40 |
| Location (Rural – reference) |  |  |  |  |  |  |
| Urban | -0.305 | -26.27 | -53.00; 15.68 | -0.129 | -12.11 | -40.10; 28.96 |
| **Facility Size Characteristics** |  |  |  |  |  |  |
| Total number of mother and child health visits per facility (at baseline in 2009) ^c^ | 0.000 | 0.00 | 0.00; 0.00 | 0.000 | 0.00 | 0.00; 0.00 |
| Total outpatient visits per year per facility ^d^ | 0.000 | 0.00 | 0.00; 0.00 | 0.000 | 0.00 | 0.00; 0.00 |
| Size of facility (total square meters of consultation rooms) ^e^ | 0.000 | 0.00 | 0.00; 0.01 | 0.000 | 0.00 | 0.00; 0.01 |
| Total staff FTE across all services per facility | -0.015 | -1.46 | -3.87; 1.00 | -0.010 | -1.02 | -2.87; 0.87 |
| Proportion of clinical staff out of total stuff per facility | -0.402 | -0.40 | -1.57; 0.77 | -0.540 | -0.54 | -1.58; 0.50 |
| **Study Selection Characteristics** |  |  |  |  |  |  |
| Intervention/Arm (Comparison – reference) |  |  |  |  |  |  |
| Intervention | 0.176 | 19.27 | -9.55; 57.26 | 0.184 | 20.22 | -4.24; 50.92 |
| Region (Central Province – reference) |  |  |  |  |  |  |
| Eastern Province | -0.322 | -27.51 | -50.31; 5.74 | -0.246 | -21.79 | -42.51; 6.39 |
| **Workload Characteristics** |  |  |  |  |  |  |
| Facility Workload (at baseline in 2009) | -0.004 | 0.00 | -0.49; 0.48 | 0.069 | 0.07 | -0.29; 0.43 |
| Time of arrival at facility | -0.001^**^ | -0.07 | -0.11; -0.02 | -0.001^**^ | -0.08 | -0.12; -0.03 |
| Waiting time ^f, g^ | 0.000 | -0.02 | -0.09; 0.06 | 0.000 | -0.05 | -0.11; 0.02 |

CIs: Confidence Intervals; FP: Family planning; HR: Human resources; OLS: Ordinary Least Squares; PITC: Provider-initiated HIV counselling and testing; PNC: Postnatal Care.

Model 1 using full range of consultation duration times, Model 2 restricted to consultation duration times of over 3 minutes.

^a^: Regression coefficients were converted into per cent change in consultation duration times using the formula [exp(β)−1]100. Proportion of clinical staff out of total stuff per facility and facility workload were log transformed and treated as elasticities.

^b^: ‘Adult’ indicates an individual going to the consultation alone, and ‘adult with child’ indicates an individual going to the consultation with a child.

^c^: Beta = -0.0000119, % change = -0.001%, 95% CIs = -0.005% to 0.002% in model 1. Beta = -0.0000137, % change = -0.001%, 95% CIs = -0.004% to 0.001% in model 2.

^d^: Beta = 0.0000018, % change = 0.0002%, 95% CIs = -0.002% to 0.002% in model 1. Beta = -0.0000024, % change = -0.0002%, 95% CIs = -0.002% to 0.001% in model 2.

^e^: Beta = 0.0000341, % change = 0.003%, 95% CIs = -0.001% to 0.008% in model 1. Beta = 0.0000267, % change = 0.003%, 95% CIs = -0.0004% to 0.0057 in model 2.

^f^: Beta = -0.0001447 in model 1. Beta = -0.0004582 in model 2.

^g^: Between arrival and first consultation (and subsequent consultations when there was more than one consultation).

^*^ *p* < 0.05, ^**^ *p* < 0.01, ^***^ *p* < 0.001
