# Supplementary material for: Impact of integration of sexual and reproductive health services on consultation duration times: results from the Integra Initiative
Source: Health Policy Plan. 2017 Nov 24;32(Suppl 4):iv82–90. doi: 10.1093/heapol/czx141 (PMC5886289; doi:10.1093/heapol/czx141)
Supplement: Supplementary Tables R2 [file czx141_tables_r2.docx]

Table 1. Summary statistics for facility characteristics (n=24).

|  | **Mean** | **SD** | **Median** | **Q1; Q3** | **Range** |
| --- | --- | --- | --- | --- | --- |
| **Integration Characteristics** |  |  |  |  |  |
| Total number of services provided per facility | 39.7 | 16.7 | 41.2 | 24.9; 51.3 | 14.2; 79.7 |
| **Facility Size Characteristics** |  |  |  |  |  |
| Total number of mother and child health visits per facility (at baseline in 2009) | 14,191 | 11,974.8 | 9,010 | 6,329; 21,686 | 2,354; 44,840 |
| Total outpatient visits per year per facility | 19,064 | 14,312.0 | 15,310 | 8,545; 21,804 | 3,805; 52,642 |
| Size of facility (total square meters of consultation rooms) | 3,409.8 | 5,233.2 | 894.5 | 513.8; 4,189.4 | 181.8; 19,542.8 |
| Total staff FTE across all services per facility | 33.9 | 14.9 | 31.6 | 21.6; 48.6 | 6.2; 55.9 |
| Proportion of clinical staff out of total stuff per facility (%) | 48.8% | 4.3% | 49.5% | 46.6%; 51.6% | 38.6%; 56.8% |
| **Workload Characteristics** |  |  |  |  |  |
| Facility workload (at baseline in 2009) (%) | 24.9% | 17.9% | 21.9% | 13.1%; 30.8% | 3.6%; 84.8% |

Table 2. Summary statistics on clients’ consultation duration times (n=3,160).

| **Variable** | **Median** | **Q1; Q3** | **Range** |
| --- | --- | --- | --- |
| **Integration Characteristics** |  |  |  |
| PITC only | 30 | 15; 53 | 1; 291 |
| FP only | 8 | 4; 16 | 1; 268 |
| Integrated PITC & FP ^a^ | 10 | 5; 21 | 1; 183 |
| Integrated PITC & FP (Joined) | 9 | 5; 18 | 1; 183 |
| Integrated PITC & FP (Separate) | 32 | 23; 44 | 6; 146 |
| **Client Characteristics** |  |  |  |
| Client type |  |  |  |
| Adult | 10 | 5; 26 | 1; 291 |
| Adult + child | 10 | 5; 21 | 1; 268 |
| Unknown | 8 | 5; 15 | 3; 103 |
| **Inpatient/Facility Characteristics** |  |  |  |
| Inpatient/Outpatient |  |  |  |
| Inpatient | 10 | 5; 20 | 1; 291 |
| Outpatient | 13 | 6; 27 | 1; 159 |
| Location |  |  |  |
| Rural | 10 | 5; 22 | 1; 268 |
| Urban | 11 | 5; 26 | 1; 291 |
| **Study Selection Characteristics** |  |  |  |
| Intervention/Arm |  |  |  |
| Comparison | 7 | 4; 15 | 1; 225 |
| Intervention | 15 | 6; 30 | 1; 291 |
| FP/PNC model |  |  |  |
| FP model | 15 | 7; 29 | 1; 256 |
| PNC model | 7 | 4; 15 | 1; 291 |
| **Workload Characteristics** |  |  |  |
| Pooled consultation duration times (mins) ^c^ | 10 | 5; 24 | 1; 291 |

FP: Family planning; PITC: Provider-initiated HIV counselling and testing; PNC: Postnatal Care; SD: Standard deviation.

^a^: Classified as integrated services when a combination of FP and PITC services were provided in a single consultation (joined) or when a combination of FP and PITC services were used in different consultations (separate). Classified as FP when at least one of the consultations was FP and none of the other consultation were PITC. Classified as PITC when at least one of the consultations was PITC and none of the other consultations were FP.

^b^: ‘Adult’ indicates an individual going to the consultation alone, and ‘adult with child’ indicates an individual going to the consultation with a child.

^c^: Pooled consultation duration times data of up to five consultations excluding any services not classified as FP, PITC or integrated PITC & FP.

Table 3. Summary statistics on clients’ consultation duration times ^a^ by type of service (n=3,160)

|  | **PITC only (n=541)** | | | **FP only (n=1,897)** | | | **Integrated PITC and FP ^b^ (n=722)** | | |
| --- | --- | --- | --- | --- | --- | --- | --- | --- | --- |
|  | Median | Q1; Q3 | Range | Median | Q1; Q3 | Range | Median | Q1; Q3 | Range |
| **Client Characteristics** |  |  |  |  |  |  |  |  |  |
| Client type ^c^ |  |  |  |  |  |  |  |  |  |
| Adult | 34 | 18; 61 | 2; 291 | 6 | 4; 14 | 1; 219 | 15 | 7; 26 | 1; 168 |
| Adult + child | 20 | 9; 32 | 1; 159 | 10 | 5; 20 | 1; 268 | 7 | 5; 17 | 1; 183 |
| Unknown | 15 | . | 15; 15 | 6 | 4; 9 | 3; 103 | 21 | 10; 32 | 10; 32 |
| **Inpatient/Facility Characteristics** |  |  |  |  |  |  |  |  |  |
| Inpatient/Outpatient |  |  |  |  |  |  |  |  |  |
| Inpatient | 27 | 11; 48 | 1; 291 | 8 | 4; 16 | 1; 268 | 7 | 5; 15 | 1; 183 |
| Outpatient | 31 | 17; 57 | 4; 159 | 8 | 4; 17 | 1; 132 | 18 | 11; 36 | 1; 147 |
| Location |  |  |  |  |  |  |  |  |  |
| Rural | 28 | 15; 55 | 1; 169 | 8 | 4; 16 | 1; 268 | 7 | 5; 16 | 1; 183 |
| Urban | 30 | 15; 51 | 2; 291 | 9 | 4; 16 | 1; 256 | 16 | 8.5 – 30 | 2; 137 |
| **Study Selection Characteristics** |  |  |  |  |  |  |  |  |  |
| Intervention/Arm |  |  |  |  |  |  |  |  |  |
| Comparison | 19 | 10; 34 | 1; 225 | 6 | 4; 13 | 1; 162 | 6 | 5; 13 | 1; 183 |
| Intervention | 37 | 22; 60.5 | 3; 291 | 10 | 5; 20 | 1; 268 | 20.5 | 12; 35 | 1; 147 |
| FP/PNC model |  |  |  |  |  |  |  |  |  |
| FP model | 27 | 14; 46 | 2; 180 | 11 | 5; 22 | 1; 256 | 18 | 10; 30 | 1; 146 |
| PNC model | 33 | 15; 68 | 1; 291 | 5 | 4; 10 | 1; 268 | 6 | 4; 12 | 1; 183 |

FP: Family planning; PITC: Provider-initiated HIV counselling and testing; PNC: Postnatal Care; SD: Standard deviation

^a^: Pooled consultation duration times data of up to five consultations excluding any services not classified as FP, PITC or integrated PITC & FP.

^b^: Classified as integrated services when a combination of FP and PITC services were provided in a single consultation or, only in the case of individual-level data, when a combination of FP and PITC services were used in different consultations. Classified as FP when at least one of the consultations was FP and none of the other consultation were PITC. Classified as PITC when at least one of the consultations was PITC and none of the other consultations were FP.

^c^: ‘Adult’ indicates an individual going to the consultation alone, and ‘adult with child’ indicates an individual going to the consultation with a child.

**Table 4. Impact of plausible determinants on log transformed minutes of clients’ consultation duration times.**

|  | **Model 1 (n=3,160)** | | | **Model 2 (n=2,929)** | | | **Model 3 (n=2,491)** | | |
| --- | --- | --- | --- | --- | --- | --- | --- | --- | --- |
| **Integration Characteristics** | **beta** | **% change**^a^ | **95% CIs**^a^ | **beta** | **% change**^a^ | **95% CIs**^a^ | **beta** | **% change**^a^ | **95% CIs**^a^ |
| Integrated PITC and FP (reference) |  |  |  |  |  |  |  |  |  |
| PITC only | 0.558^***^ | 74.72 | 39.70; 118.40 | 0.485^***^ | 62.47 | 30.09; 102.90 | 0.018 | 1.84 | -20.98; 31.24 |
| FP only | -0.566^***^ | -43.22 | -54.34; -29.36 | -0.509^***^ | -39.87 | -50.42; -27.08 | -1.106^***^ | -66.90 | -75.81; -54.70 |
| Total number of services provided per facility | -0.007 | -0.70 | -1.48; 0.01 | -0.009^*^ | -0.91 | -1.58; -0.24 | -0.003 | -0.32 | -1.06; 0.42 |
| **Client Characteristics** |  |  |  |  |  |  |  |  |  |
| Age (years) | -0.006^*^ | -0.60 | -1.09; -0.09 | -0.004 | -0.41 | -0.93; 0.10 | -0.007^*^ | -0.68 | -1.23; -0.12 |
| Client type ^b^ (Adult – reference) |  |  |  |  |  |  |  |  |  |
| Adult + child | 0.042 | 4.29 | -9.99; 20.77 | 0.029 | 2.97 | -9.52; 17.18 | 0.066 | 6.87 | -7.28; 23.19 |
| Unknown | -0.241 | -21.41 | -51.04; 26.01 | -0.355 | -29.90 | -56.70; 13.49 | -0.350 | -29.56 | -59.05; 21.18 |
| **Inpatient/Facility Characteristics** |  |  |  |  |  |  |  |  |  |
| Inpatient (Reference) |  |  |  |  |  |  |  |  |  |
| Outpatient | 0.129 | 13.77 | -17.98; 57.82 | 0.093 | 9.70 | -17.51; 45.88 | 0.112 | 11.81 | -19.58; 55.45 |
| Location (Rural – reference) |  |  |  |  |  |  |  |  |  |
| Urban | 0.535^*^ | 70.74 | 1.93; 186.04 | 0.369 | 44.70 | -5.13; 120.70 | 0.385 | 46.93 | -16.17; 157.53 |
| **Facility Size Characteristics** |  |  |  |  |  |  |  |  |  |
| Total number of mother and child health (MCH) visits per facility (at baseline in 2009) ^c^ | 0.000 | 0.00 | -0.01; 0.00 | 0.000 | 0.00 | -0.01; 0.00 | 0.000 | 0.00 | -0.01; 0.00 |
| Total outpatient visits per year per facility ^d^ | 0.000 | 0.00 | 0.00; 0.00 | 0.000 | 0.00 | 0.00; 0.00 | 0.000 | 0.00 | 0.00; 0.00 |
| Size of facility (total square meters of consultation rooms) ^e^ | 0.000 | 0.00 | 0.00; 0.01 | 0.000 | 0.00 | 0.00; 0.01 | 0.000 | 0.00 | 0.00; 0.01 |
| Total staff FTE across all services per facility | -0.007 | -0.70 | -1.97; 0.51 | -0.007 | -0.72 | -1.81; 0.39 | -0.005 | -0.54 | -1.97; 0.92 |
| Proportion of clinical staff out of total stuff per facility | -3.920^**^ | -98.02 | -99.80; -80.23 | -3.548^**^ | -97.12 | -99.63; -77.62 | -3.828^**^ | -97.83 | -99.80; -76.18 |
| **Study Selection Characteristics** |  |  |  |  |  |  |  |  |  |
| Intervention/Arm (Comparison – reference) |  |  |  |  |  |  |  |  |  |
| Intervention | 0.396^**^ | 48.59 | 18.56; 86.31 | 0.353^**^ | 42.37 | 16.07; 74.62 | 0.315^*^ | 36.99 | 7.15; 75.14 |
| FP/PNC (FP model – reference) |  |  |  |  |  |  |  |  |  |
| PNC model | -0.367^*^ | -30.72 | -51.65; -0.82 | -0.283 | -24.63 | -43.74; 0.96 | -0.331 | -28.15 | -51.70; 6.88 |
| **Workload Characteristics** |  |  |  |  |  |  |  |  |  |
| Facility Workload (at baseline in 2009) | 1.189 | 228.38 | -4.21; 1026.47 | 0.992^*^ | 169.53 | 3.28; 603.43 | 1.132 | 210.14 | -7.10; 935.44 |
| Time of arrival at facility | -0.001^**^ | -0.10 | -0.12; -0.02 | -0.001^***^ | -0.08 | -0.12; -0.04 | -0.001^*^ | -0.07 | -0.13; -0.01 |
| Waiting time ^f, g^ | 0.000 | 0.00 | -0.09; 0.09 | -0.000 | -0.01 | -0.09; 0.06 | 0.000 | 0.03 | -0.07; 0.13 |
| **R^2^** | 0.309 |  |  | 0.311 |  |  | 0.307 |  |  |
| **AIC** | 8,208 |  |  | 6,999 |  |  | 6,603 |  |  |
| **BIC** | 8,323 |  |  | 7,113 |  |  | 6,714 |  |  |

AIC: Akaike Information Criterion; BIC: Bayesian Information Criterion; CIs: Confidence Intervals; FP: Family planning; HR: Human resources; OLS: Ordinary Least Squares; PITC: Provider-initiated HIV counselling and testing; PNC: Postnatal Care.

Model 1 using full range of consultation duration times, Model 2 restricted to consultation duration times of over 3 minutes; Model 3 restricted to separate consultations only.

^a^: Regression coefficients were converted into per cent change in consultation duration times using the formula [exp(β)−1]100.

^b^: ‘Adult’ indicates an individual going to the consultation alone, and ‘adult with child’ indicates an individual going to the consultation with a child.

^c^: Beta = -0.0000317, % change = -0.003%, 95% CIs = -0.0068% to 0.0005% in model 1. Beta = -0.0000263, % change = -0.003%, 95% CIs = -0.0055% to 0.0002% in model 2.

^d^: Beta = -0.0000072, % change = -0.001%, 95% CIs = -0.003% to 0.001% in model 1. Beta = -0.00000631, % change = -0.001%, 95% CIs = -0.002% to 0.001% in model 2.

^e^: Beta = 0.0000373, % change = 0.004%, 95% CIs = -0.001% to 0.009% in model 1. Beta = 0.0000333, % change = 0.003%, 95% CIs = -0.001% to 0.007% in model 2.

^f^: Beta = 0.0000065, % change = 0.001% in model 1. Beta = -0.0001351, % change = -0.001%, 95% CIs = -0.004% to 0.001% in model 2.

^g^: Between arrival and first consultation (and subsequent consultations when there was more than one consultation).

^*^ *p* < 0.05, ^**^ *p* < 0.01, ^***^ *p* < 0.001.
